# *TOPGEAR*: a randomised phase III trial of perioperative ECF chemotherapy versus preoperative chemoradiation plus perioperative ECF chemotherapy for resectable gastric cancer (an international, intergroup trial of the AGITG/TROG/EORTC/NCIC CTG)

**DOI:** 10.1186/s12885-015-1529-x

**Published:** 2015-07-21

**Authors:** Trevor Leong, B Mark Smithers, Michael Michael, Val Gebski, Alex Boussioutas, Danielle Miller, John Simes, John Zalcberg, Karin Haustermans, Florian Lordick, Christoph Schuhmacher, Carol Swallow, Gail Darling, Rebecca Wong

**Affiliations:** 1 Peter MacCallum Cancer Centre, Locked Bag 1, A’Beckett Street, Victoria, 8006 Australia; 2 Upper Gastro-intestinal and Soft Tissue Unit, School of Medicine, University of Queensland, Princess Alexandra Hospital, Ipswich Road, Woolloongabba, QLD 4102 Australia; 3 NHMRC Clinical Trials Centre, University of Sydney, Locked bag 77, Camperdown, NSW 1450 Australia; 4 School of Public Health and Preventive Medicine, Monash University, Level 5 The Alfred Centre, 99 Commercial Road, Melbourne, VIC 3004 Australia; 5 Radiation Oncology, University Hospitals Leuven, Department of Oncology, KU, Leuven, Belgium; 6 University Cancer Center Leipzig (UCCL), University Medicine Leipzig, Leipzig, Germany; 7 European Clinical Research Infrastructure Network, ECRIN-ERIC, Paris Biopark, 5 rue Watt, 75013 Paris, France; 8 Mount Sinai Hospital, Rm 1225, 600 University Avenue, Toronto, ON M5G 1X5 Canada; 9 Toronto General Hospital, 9N-955, 200 Elizabeth St, Toronto, ON M5G 2C4 Canada; 10 Princess Margaret Hospital, Rm 5-807, 610 University Avenue, Toronto, M5G 2M9 Canada

**Keywords:** Gastric cancer, Preoperative chemoradiotherapy, Perioperative chemotherapy, Adjuvant therapy

## Abstract

**Background:**

The optimal management of patients with resectable gastric cancer continues to evolve in Western countries. Following publication of the US Intergroup 0116 and UK Medical Research Council MAGIC trials, there are now two standards of care for adjuvant therapy in resectable gastric cancer, at least in the Western world: postoperative chemoradiotherapy and perioperative epirubicin/cisplatin/fluorouracil (ECF) chemotherapy.

We hypothesize that adding chemoradiation to standard perioperative ECF chemotherapy will achieve further survival gains. We also believe there are advantages to administering chemoradiation in the preoperative rather than postoperative setting. In this article, we describe the *TOPGEAR* trial, which is a randomised phase III trial comparing control arm therapy of perioperative ECF chemotherapy with experimental arm therapy of preoperative chemoradiation plus perioperative ECF chemotherapy.

**Methods/Design:**

Eligible patients with resectable adenocarcinoma of the stomach or gastroesophageal junction will be randomized to receive either perioperative chemotherapy alone (3 preoperative and 3 postoperative cycles of ECF) or perioperative chemotherapy plus preoperative chemoradiation. In the chemoradiation arm, patients receive 2 cycles of ECF plus chemoradiation prior to surgery, and then following surgery 3 further cycles of ECF are given.

The trial is being conducted in two Parts; Part 1 (phase II component) has recruited 120 patients with the aim of assessing feasibility, safety and preliminary efficacy of preoperative chemoradiation. Part 2 (phase III component) will recruit a further 632 patients to provide a total sample size of 752 patients. The primary endpoint of the phase III trial is overall survival. The trial includes quality of life and biological substudies, as well as a health economic evaluation. In addition, the trial incorporates a rigorous quality assurance program that includes real time central review of radiotherapy plans and central review of surgical technique.

**Discussion:**

*TOPGEAR* is an international, intergroup collaboration led by the Australasian Gastro-Intestinal Trials Group (AGITG), in collaboration with the Trans-Tasman Radiation Oncology Group (TROG), European Organisation for Research and Treatment of Cancer (EORTC) and the NCIC Clinical Trials Group. It addresses a globally significant question that will help inform future international standards for clinical practice in resectable gastric cancer.

**Trial registration:**

Australian New Zealand Clinical Trials Registry: ACTRN12609000035224. Registered 30 May 2009

## Background

The optimal management of patients with resectable gastric cancer continues to evolve in Western countries. Following publication of the US Intergroup trial 0116 in 2001, postoperative chemoradiation became a standard of care for patients who had undergone potentially curative surgery [1]. This trial randomly assigned 556 patients following surgery to either observation or adjuvant therapy with 4 monthly cycles of bolus 5-fluorouracil (5-FU) and leucovorin combined with 45 Gy of radiotherapy. With a median follow-up of 5 years, the 3-year survival rate was 50 % in the chemoradiation group versus 41 % in the surgery alone group (p = 0.005). Although adjuvant chemoradiotherapy has been adopted as standard of care in North America and some parts of the world, it is still uncommonly used in other countries. This relates mainly to criticism of INT0116 with regard to surgical quality as 54 % of patients underwent less than a D1 lymph node dissection despite the recommendation for a D2 dissection. There are also concerns amongst medical and radiation oncologists regarding the outdated chemoradiation regimen that was employed.

The UK Medical Research Council MAGIC trial that was published in 2006 provides an alternative standard of care for adjuvant therapy in resectable gastric cancer [2]. This trial randomly assigned 503 patients with resectable gastric cancer to either perioperative chemotherapy and surgery or surgery alone. Chemotherapy consisted of 3 preoperative and 3 postoperative cycles of epirubicin, cisplatin and 5-FU (ECF). With a median follow-up of 4 years, the 5-year survival rate was 36 % in the perioperative chemotherapy group versus 23 % in the surgery alone group (hazard ratio 0.75; p = 0.009). A significantly greater proportion of patients in the perioperative chemotherapy group underwent curative R0 resection (79 % vs 70 %), and resected tumors were significantly smaller and less advanced in this group. Of those patients who started chemotherapy, 90.7 % completed preoperative chemotherapy. However, of those who completed preoperative chemotherapy and surgery, only 49.5 % also completed postoperative chemotherapy.

Since the publication of the INT0116 and MAGIC studies, clinicians and patients have been faced with the dilemma of which adjuvant or neoadjuvant strategy to employ. By analysing failure patterns, each approach appears to improve survival through different mechanisms. The perioperative chemotherapy approach using ECF reduces systemic failure, while postoperative chemoradiation improves locoregional control. Since both strategies provide moderate gains in survival, we hypothesize that adding chemoradiation to standard perioperative ECF chemotherapy will achieve even greater survival gains in a similar patient population. Furthermore, we believe there are advantages to testing the addition of chemoradiation by administering it in the preoperative rather than postoperative setting. One of the main advantages of preoperative therapy is the potential for tumor downstaging with an increase in the complete R0 resection rate. Preoperative therapy is also much better tolerated than postoperative therapy, thereby ensuring that all patients receive the intended treatment. Both of these advantages were clearly demonstrated in the MAGIC study. The strategy of preoperative chemoradiation for gastric cancer has thus far only been tested in a small number of phase II studies, which have demonstrated safety, tolerability and high rates of pathological complete response [3, 4].

In this article, we describe the study protocol of a currently accruing trial, which has the acronym *TOPGEAR* (Trial Of Preoperative therapy for Gastric and Esophagogastric junction AdenocaRcinoma). *TOPGEAR* is a randomised phase III trial, which compares control arm therapy of perioperative ECF chemotherapy with experimental arm therapy of preoperative chemoradiation plus perioperative ECF chemotherapy. The design of this trial allows a comparison of the MAGIC regimen with an INT0116 regimen, but with the specific intent of bringing the chemoradiation approach into the preoperative setting. This trial is an international, intergroup collaboration led by the Australasian Gastro-Intestinal Trials Group (AGITG), in collaboration with the Trans-Tasman Radiation Oncology Group (TROG), European Organisation for Research and Treatment of Cancer (EORTC) and the NCIC Clinical Trials Group.

## Methods/design

### Design

**Fig. 1:**
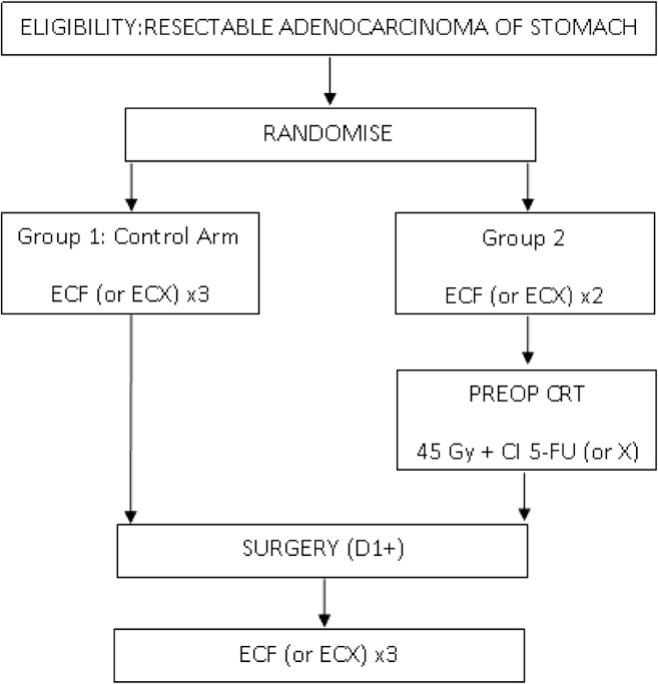
Trial schema. Patients are randomised to either perioperative chemotherapy alone or perioperative chemotherapy plus preoperative chemoradiation (preop CRT). X = capecitabine

*TOPGEAR* is a two arm randomised phase II/III trial in which patients are randomised to either perioperative chemotherapy alone (3 preoperative and 3 postoperative cycles of ECF) or perioperative chemotherapy plus preoperative chemoradiation (Fig. 1). In the chemoradiation arm, patients receive 2 cycles of ECF plus chemoradiation prior to surgery, and then following surgery 3 further cycles of ECF are given. The method of randomisation will be using minimisation where patients will be stratified on age, primary tumor site, clinical tumor stage, nodal status, treating institution, and staging investigations. The protocol has been approved by the Clinical Research Ethics Committee of the Cancer Institute NSW, as well as individual institutional ethics committees. All patients have given written informed consent before participating in the trial.

The primary objective of this phase III trial is to investigate whether perioperative chemotherapy plus preoperative chemoradiation improves overall survival compared to perioperative chemotherapy alone. The trial will be conducted in two Parts using an adaptive phase II → III design. Part 1 (phase II component) has recruited 120 patients with the aim of assessing feasibility, safety and preliminary efficacy of preoperative chemoradiation. The endpoints for Part 1 are pathological complete response (pCR) rate, toxicity, treatment compliance and accrual. If based on an interim analysis of the first 120 patients, the experimental chemoradiation arm is shown to be safe and feasible, accrual is deemed to be satisfactory, and there is not clear evidence of lack of efficacy as reviewed by the Independent Data and Safety Monitoring Committee (IDSMC), recruitment will continue in the second Part of the study (phase III component), which will recruit a further 632 patients for a total of 752 (recruitment to Part 2 will begin prior to this interim analysis in a seamless transition). The primary endpoint of Part 2 will be overall survival, with progression-free survival, toxicity, and pathological response rate as secondary endpoints.

### Participants

Eligible patients are those who meet all of the following eligibility criteria:

1. Histologically proven adenocarcinoma of the stomach or gastroesophageal junction that is:
  - Stage IB (T1N1 only, T2N0 not eligible) – IIIC, i.e. T3 – T4 and/or N + ve, according to AJCC 7th edition.
  - Considered operable following initial staging investigations (surgeon believes that an R0 resection can be achieved).
    (Gastroesophageal tumors are defined as tumors that arise in the cardia or at the gastroesophageal junction that do not involve more than 2 cm of the lower esophagus, i.e. Siewert Type II and Siewert Type III)
2. Age 18 years or older
3. Eastern Cooperative Oncology Group (ECOG) performance status 0–1
4. Adequate organ function defined as follows:
  Bone marrow: Hemoglobin ≥ 90 g/L, Absolute neutrophil count (ANC) ≥ 1.5 x 10^9^ /L, White blood cell count ≥ 3 x 10^9^ /L, Platelet count ≥ 100 x 10^9^ /L
  Hepatic: Serum bilirubin ≤ 1.5 x ULN, AST and/or ALT ≤ 3.0 x ULN
  Renal: Serum creatinine ≤ 0.150 mmol/L, and calculated creatinine clearance ≥ 50 mL/min
5. Any patient with a history of ischaemic heart disease and abnormal ECG, or who is over 60 years of age should have a pre-treatment evaluation of cardiac function with a MUGA scan or echocardiogram. Patients will only be included if the left ventricular ejection fraction is ≥ 50 %.

### Study treatments

#### Pre- and post-operative chemotherapy

ECF consisting of:

- Epirubicin 50 mg/m^2^ IV day 1
- Cisplatin 60 mg/m^2^ IV day 1
- 5-FU 200 mg/m^2^/day IV via 21 day continuous infusion. According to centre specific preferences, capecitabine 625 mg/m^2^ bid days 1–21 may be substituted for 5-FU. This regimen is named “ECX”. For simplicity, the text will refer only to ECF.

#### Chemoradiotherapy

Radiation therapy consisting of 45 Gy in 25 fractions, 5 days per week for 5 weeks + continuous infusional 5-FU 200 mg/m^2^/day, 7 days per week throughout the entire period of radiotherapy (or capecitabine 825 mg/m^2^ bid, days 1 to 5 each week of RT). Radiation therapy will be delivered to the entire stomach, any perigastric tumor extension, and regional lymph nodes according to detailed guidelines contained in the protocol.

#### Surgery

Surgical guidelines have been developed by the Surgical Subcommittee comprising surgeons from Australia, Europe and Canada in accordance with international standards. The acceptable resections include a total gastrectomy, a subtotal distal gastrectomy or an esophago-gastrectomy (for gastroesophageal junction tumors). The recommended operation is a D2 gastrectomy where possible, with a minimum approach being a D1+ gastrectomy aiming for complete resection of the primary cancer and its draining nodes.

The full trial protocol includes considerably more detail about chemotherapy concurrent medications and dose attenuations, radiation specifics (including contouring atlas) and surgery details.

### Quality assurance

The trial includes a comprehensive quality assurance program to ensure patient safety, appropriate trial conduct and data quality, including:

#### Surgical quality assurance

There is central review of surgical technique to ensure compliance with protocol guidelines. Each surgeon completes a trial operation form indicating the extent of surgery, the lymph node stations resected and the reconstruction. The form is reviewed by a Surgical Subcommittee, which will make an independent assessment of the extent of lymphadenectomy. In addition, the pathology will be reviewed and used as a surrogate for adequacy of resection. It is expected that at least 15 lymph nodes will be resected during a radical gastrectomy for cancer. Additionally, the Maruyama Index (MI) [5] will be assessed for each specimen. A low MI suggests an adequate lymphadenectomy and is associated with increased survival when compared with a high MI; this provides a second objective measure assessing surgical quality assurance in each group. Because of the importance of pathological assessment, the trial also incorporates a process of central pathology review.

#### Radiation therapy quality assurance (RTQA)

Because of the complexity of gastric irradiation, a comprehensive RTQA program has been developed to monitor protocol violations. The technical review will be conducted at 2 time points; pre-radiation therapy and post-treatment. The pre-treatment review will be conducted in “real time” - at least 1 week before radiation therapy is to begin, to allow any adjustments to be made. Approval of the treatment plan must be obtained prior to the patient starting radiotherapy. The main parameters to be evaluated will be clinical target volume (CTV) coverage and dose to organs at risk (OAR). An analysis of INT0116 demonstrated that 35 % of treatment plans contained protocol violations at initial pre-treatment review. The post-treatment review will assess compliance of radiotherapy delivery with protocol guidelines after completion of radiotherapy. The RTQA process will include the first 5 patients from each treatment centre; once an acceptable quality level is achieved, sites will be audited with one in every three patients.

### Substudies

#### Biological substudy

In addition to addressing clinical questions, the trial also incorporates a comprehensive biological research program. Blood, serum, plasma and tumor specimens will be biobanked. Tumor specimens will include material from pre-treatment biopsies and gastric resections. In addition to formalin-fixed / paraffin-embedded (FFPE) material, fresh frozen tissue (FFT) collection will be undertaken at a subset of Australian and Canadian centres. This will provide the potential for discovery using FFT specimens and validation with FFPE specimens. Broad objectives of the translational research program are to create a tissue bank of well curated biospecimens with coded clinical annotations and to undertake both discovery/hypothesis-generation and validation of existing hypotheses addressing the questions; “Are there genetic determinants of chemotherapeutic and radiotherapeutic response in gastric cancer?”, and “Are there biological differences in remnant disease after preoperative chemoradiation that differ from the primary (treatment naïve) lesion that could be used to target specific therapeutics?” In addition, the program will investigate a number of specific biomarkers identified *a priori*, which focus on both discovery/hypothesis generation and validation objectives.

#### Quality of life (QoL) and economic evaluation

This study will collect and assess QoL data for a large group of patients receiving different adjuvant treatments. QoL in this study will be assessed using the EORTC QLQ-C30 core questionnaire as well as the QLQ-OG25 module, which has been developed to measure QoL in patients with cancer of the esophagus, the gastroesophageal junction and the stomach. We hypothesize that perioperative chemotherapy plus preoperative chemoradiation will improve disease control and overall survival but may also be associated with greater treatment-related morbidity. Thus, related to the patient’s experience, a preference-based health status will also be collected for a subset of patients. The Health Utilities Index Mark3 (HUI3) will be used as an indirect measure of health preference; the HUI3 is a health-related QoL instrument and will be used to help estimate quality-adjusted life years (QALYs) for each intervention. A health economic assessment will be undertaken utilising information on QoL related to initial treatment, progression, overall survival and initial resource usage including treatment delivery as inputs.

### Statistical considerations

The sample size of the randomised phase III trial has been planned to ensure sufficient power to demonstrate an overall survival advantage of perioperative chemotherapy plus preoperative chemoradiation as compared with perioperative chemotherapy alone. The expected 5-year survival for perioperative chemotherapy alone is approximately 40 % and is supported by the observations from the MAGIC trial (5-year survival 36 %) [2]. We would consider an approximate 25 % relative increase in this rate (hazard ratio [HR] = 0.76) to be clinically worthwhile and feasible; this translates to an estimated 5-year survival of 50 %. The potential to achieve this incremental benefit is suggested by 3 pieces of information: i) the HR observed in the INT0116 trial with postoperative CRT was 0.74 [1]; ii) the odds ratio [OR] for 5-year survival observed in a meta-analysis of 3 trials comparing postoperative chemoradiation to surgery +/− chemotherapy was 0.45 [6]; and, iii) the OR for 5-year survival observed in a meta-analysis of 4 trials comparing preoperative radiation therapy to surgery alone was 0.62 [6, 7]. The objective of this randomised phase III trial is to determine whether these benefits of radiation therapy, especially when given in its most optimal form and timing of preoperative chemoradiation, translate into the same magnitude of benefit when patients receive perioperative chemotherapy. With 80 % power and 95 % confidence, 410 deaths need to be observed. We determine that this event number would be observed by accruing 720 patients over 5 years with an additional follow-up of 3 years. Allowing for a dropout rate of 7 %, accrual of 752 patients is required.

The differences in overall survival between the two treatment arms will be compared using an unadjusted two sided log-rank test at the p = 0.05 significance level (α = 0.05). Kaplan-Meier curves will be displayed. Survival will be characterized in terms of the median, and the probability of being alive at 6 months and at 12 months (based on Kaplan-Meier estimates). Ranges, 95 % confidence intervals on the treatment estimates, and the hazard ratio (estimated by Cox proportional hazards regression) will also be computed. Exploratory multivariable modelling will be performed adjusting for baseline levels of primary tumor site, age, gender nodal status and clinical tumor stage. The analyses will be performed on an intention to treat basis.

## Discussion

The *TOPGEAR* trial addresses a globally significant question that will help inform future international standards for clinical practice in resectable gastric cancer. Since the reporting of the INT0116 and MAGIC trials, there have been no published randomized trials directly comparing these two approaches, each of which was found to be superior to surgery alone; nor have there been any randomized controlled trials evaluating the role of preoperative chemoradiation. A recently completed US Intergroup trial (CALGB 80101) compared the superior arm from INT0116 (in which the chemotherapy was based on 5-FU) with a postoperative chemoradiation regimen that employed postoperative ECF as the systemic chemotherapy component. As both arms of CALGB 80101utilized postoperative chemoradiation, this trial was not able to assess the potential benefits of preoperative chemotherapy and radiotherapy. The ongoing UK MRC trial (ST03) is a randomized trial evaluating the perioperative ECF regimen with or without bevacizumab. The potential role of chemoradiation is not an objective of this study. The ongoing Dutch CRITICS trial is a randomized trial comparing the perioperative ECF regimen with preoperative chemotherapy plus postoperative chemoradiation. CRITICS will evaluate the potential benefit of adding postoperative chemoradiation to perioperative chemotherapy.

*TOPGEAR* is the first multinational, intergroup trial addressing adjuvant therapy for gastric cancer, including the role of preoperative chemoradiation. When all sites are activated, the *TOPGEAR* research network will comprise approximately 75 centres spanning 15 countries in the Asia-Pacific region, Europe and North America. Part 1 has been completed and the IDSMC recommended that the trial should proceed to Part 2 as planned. Current accrual is 170 patients recruited from 55 active sites.
